# Artifacts in Simultaneous hdEEG/fMRI Imaging: A Nonlinear Dimensionality Reduction Approach †

**DOI:** 10.3390/s19204454

**Published:** 2019-10-14

**Authors:** Marek Piorecky, Vlastimil Koudelka, Jan Strobl, Martin Brunovsky, Vladimir Krajca

**Affiliations:** 1 National Institute of Mental Health, 25067 Klecany, Czech Republic; vlastimil.koudelka@nudz.cz (V.K.); jan.strobl@nudz.cz (J.S.); martin.brunovsky@nudz.cz (M.B.); 2 Dep. of Biomedical Technology, Faculty of Biomedical Engineering, CTU in Prague, 27201 Prague, Czech Republic; krajcvla@fbmi.cvut.cz; 3 Third Faculty of Medicine, Charles University, 10000 Prague, Czech Republic

**Keywords:** independent component analysis, hdEEG, fMRI, simultaneous measurement, artifact, nonlinear dimension reduction

## Abstract

Simultaneous recordings of electroencephalogram (EEG) and functional magnetic resonance imaging (fMRI) are at the forefront of technologies of interest to physicians and scientists because they combine the benefits of both modalities—better time resolution (hdEEG) and space resolution (fMRI). However, EEG measurements in the scanner contain an electromagnetic field that is induced in leads as a result of gradient switching slight head movements and vibrations, and it is corrupted by changes in the measured potential because of the Hall phenomenon. The aim of this study is to design and test a methodology for inspecting hidden EEG structures with respect to artifacts. We propose a top-down strategy to obtain additional information that is not visible in a single recording. The time-domain independent component analysis algorithm was employed to obtain independent components and spatial weights. A nonlinear dimension reduction technique t-distributed stochastic neighbor embedding was used to create low-dimensional space, which was then partitioned using the density-based spatial clustering of applications with noise (DBSCAN). The relationships between the found data structure and the used criteria were investigated. As a result, we were able to extract information from the data structure regarding electrooculographic, electrocardiographic, electromyographic and gradient artifacts. This new methodology could facilitate the identification of artifacts and their residues from simultaneous EEG in fMRI.

## 1. Introduction

Simultaneous electroencephalogram/functional magnetic resonance imaging (EEG/fMRI), which is experiencing a great boom in neuroscience and medicine, has delivered a new category of artifact that has to be taken into account by experts [1,2]. Generally, artifacts are signals that do not originate in the brain. While fMRI images are negligibly affected by EEG, the MRI scanner noise and magnetic field strongly influence EEG signals [2]. EEG artifacts arise from the strong magnetic field within the scanner (e.g., movement, cardioballistic artifacts (pulsion artifacts, (PAs)) [3]) and rapid changes in gradient magnetic fields during the scan (e.g., gradient artifact (GA) [4]). Instruments that are essential for operating the scanner (ventilation, helium pump [5], lights in the scanner room) add additional electromagnetic noise.

Previous studies ([6,7]) were motivated to automatically find artifacts in EEG using only the data structure (no user input). Chaumon et al., published a guide to the selection of independent components (ICs) [8] and, at the same time, presented automated selection tools for IC recognition [8]. Another tool is empirical mode decomposition (EMD), which is suitable for processing nonlinear signals and it has been used for processing biosignals [9]. Supervised learning algorithms have also been applied in the MARA algorithm [10], CORRMAP [11] and methods in Reference [12]. All these algorithms are based on decisions made by the user, who may not always respect the internal structure of the data and supervised algorithms usually suffer from poor generalization due to high inter-individual variances in brain activity [13]. A comprehensive study [8] was motivated by the fact that automated methods for isolating artifacts without supervision appear to be more appropriate and had not yet been suggested. The application of machine learning techniques for EEG physiological feature learning was presented in Reference [14].

All of the above-mentioned methods focus on EEG recordings out of the MRI scanner. Our proposed approach consists of three parts—standard EEG signal preprocessing (filtering, routine artifact suppression), the core of the method and interpretation of the resulting reduced space. By default, the EEG signal is processed by bandpass filter, which may be followed by demeaning or detrending. In the next step, automatic filters are used to suppress the artifacts that we expect in the signal. Sections that include extreme values of amplitude are routinely excluded from IC analysis (ICA).

The core of our method lies in the utilization of an unsupervised manifold learning technique, which is a novel contribution. The proposed technique is not trained on expert data annotation; instead, it is based on the internal data structure.

In general, the proposed technique searches for clusters without any prior assumption about their meaning. Thus, not all found regularities in the data represent relevant information about the dataset. Therefore, interpretation of the clinical significance of clusters is needed. It is a supervised and partially subjective process with respect to the expert who describes the data. Preprocessing and interpretation are therefore supervised. For this reason, the method is designated semi-supervised.

The presented method does not rely on any predefined features or expectations of the sensing of artifacts in the data. This allows us to observe clusters of unexpected artifacts in the data, such as the residuals of commonly used artifact correction methods.

The presented work is motivated by previous results on IC feature extraction from EEG recorded in Faraday’s cage out of the scanner (see Reference [15]). However, the current study identifies artifacts commonly found in EEG, as well as those caused by the MRI environment. Typically, two major artifacts are caused by a magnetic resonance run—the gradient artifact and cardioballistic artifact [1,4]. EEG data can also be contaminated by artifacts that arise from ventilators or interference from the helium pump used for fMRI cooling [16]. To the best of our knowledge, no method that exploits machine learning for EEG during fMRI IC identification has been published.

This manuscript contains three main parts. The first part describes the acquisition system, the origin of the dataset, an ethical statement and EEG preprocessing steps. The second part summarizes the methods used in the data analysis and describes the methodology’s core of layer extraction from hidden EEG space. The third part presents the results obtained in all stages of the analysis. The obtained clusters of ICs are also interpreted and the results are discussed in more detail in the context of neuroimaging.

## 2. Materials and Methods

In total, 54 datasets were evaluated. The datasets were gathered from simultaneously recorded High Density EEG (hdEEG) during fMRI sessions. hdEEG is used to achieve good spatial resolution and it also allows for the interpolation of floating electrodes with higher portability. The dataset consists of recordings from 44 healthy subjects and 10 patients with sleep disorders. Five different protocols are included in the dataset—awake resting state, sleep, visual stimulation, auditory stimulation and cognitive task. The different protocols suppress the influence of the physiological component and the recording methodology. The recordings vary in their length and various studies were used for significant diversity in the data.

**Figure 1:**
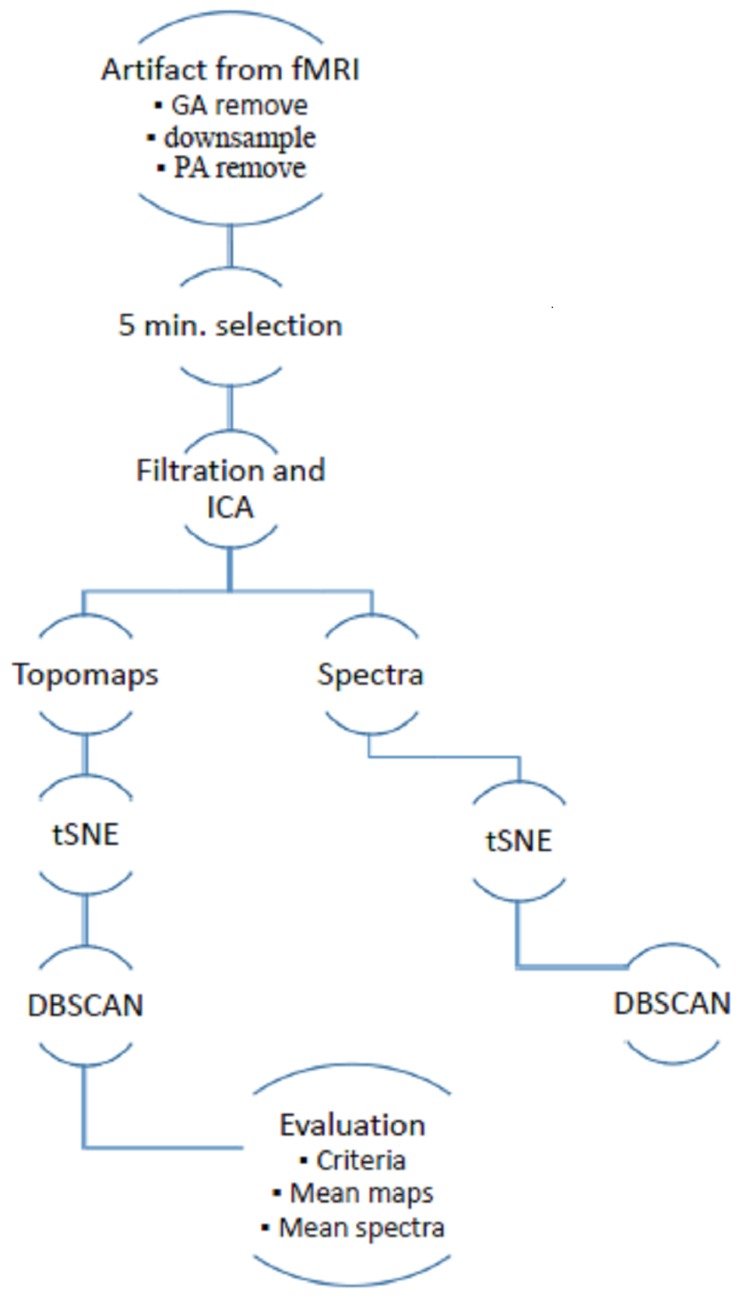
Schema of semiautomatic extraction of artifacts from simultaneously recorded electroencephalogram (EEG) in functional magentic resonance imagin (fMRI).

Data acquisition was approved by the local ethical committee of the National Institute of Mental Health, Czech Republic. All subjects involved in the study signed an informed consent form. Data were recorded using a Siemens Prisma 3T, EGI256-channel EEG system equipped by a Net Amps 400 series amplifier, $F_{s}=1000$ Hz, DC coupling with 256 HydroCel Geodesic Sensor Net and Net Station 5 acquisition software. The method was implemented in the framework of the FieldTrip software toolbox (version 20181231) and SPM8 (version April 2009) in MATLAB 2015a [13,17]. The data processing schema is shown in Figure 1. The implemented methodology is based on previous research on artifacts with non-simultaneously recorded EEG [15] and includes an extension of a temporal–spatial–frequency evaluation.

The following overview summarizes the methodology:

1. Data were preprocessed by state-of-the-art methods to first eliminate most of the variance in the artifacts.
2. Through ICA, 5-min sections of the signal were selected. A time-domain ICA algorithm was employed to obtain independent components and spatial weights. In addition, spatial weights and Fourier spectra were normalized with respect to their Root Mean Square (RMS) value.
3. We reduced the topomap space to two dimensions for better visual inspection. Multidimensional space is demanding for automatic clustering and it is not possible to have optical control of separated clusters. The nonlinear method t-SNE (t-distributed stochastic neighbor embedding) should respect the original layout of the EEG space.
4. The DBSCAN algorithm (density-based spatial clustering of applications with noise) was used to cluster the 2D space (the reduced space of topographic maps). This algorithm does not require input for the number of clusters that are not primarily visible in hidden structures. The DBSCAN algorithm determines this parameter itself and is able to separate the nested clusters.
5. We applied several criteria (autocorrelation, focal topography and focal trial activity) to the original dataset and detected whether certain criteria matched some clusters. In this way, it was possible to identify a criterion that would be suitable for describing a particular artifact.
6. The median and mean of topomaps, Fourier spectra and time series were investigated to generalize cluster patterns. These characteristics allowed for the assignment of the activity to individual artifacts.

### 2.1. Data Preprocessing

Gradient artifacts (GAs) that were triggered by the acquisition of fMRI scans were removed by the fMRI artifact slice template removal (FASTR) method [2]. The GA residue caused by initial dummy scans was removed manually from the time series. Hermite polynomials were used to down-sample the EEG signal from 1 kHz to 250 Hz after GA removal. The raw data were inspected and artifacts that would significantly affect adaptive Optimal Basis Set (AOBS analysis were rejected. The electrocardiographic (ECG) channel was used to trigger cardioballistic artifact detection by the AOBS method [18]. Poor time-series intervals with high variance were identified by Tukey’s method; these time series were rejected and then interpolated by the weighted average of all neighbors. Signals were bandpass-filtered by a two-way zero-phase finite impulse response FIR filter. The low critical frequency was $F_{low}=0.5$ Hz and the high critical frequency was $F_{high}=100.0$ Hz (see Reference [7]).

The amount of data for further IC analyses was reduced by determining 150 sections that were uniformly distributed over the whole recording. A 2-s long segment was randomly selected from each section. Thus, 5 min were selected from each EEG record.

The above-mentioned procedure is commonly used for cleaning EEG/fMRI data. In the next sections, we focus on capturing GA and PA residuals and other possible artifacts in the data.

### 2.2. ICA and Normalization

The ICA (independent component analysis) method was used to isolate statistically independent information in each EEG recording. The ICA method assumes that the EEG signal measured by electrodes is a linear combination of statistically independent sources:

(1) X=A·S,

where *X* is the matrix of the signals from the surface EEG electrodes. *A* is the mixing matrix, which determines the mixing of EEG sources as they pass through the environment and *S* is the matrix of sources.

The ICA estimates the mixing matrix *A*. We can then invert *A* and estimate the independent sources, also called components, of the EEG signal (see Equation (2)).

(2) $$Z=A^{-1}\cdot X.$$

The $A^{-1}$ matrix contains raw vectors of inverse weights, which are denoted by *W*. *Z* represents an estimate of the sources (origin sources *S*). Each particular inverse weight vector contains information about the extent to which a particular independent component explains a signal on the electrodes. In other words, the inverse weights define a spatial pattern of components. Thus, spatial patterns of independent components are contained in the $A^{-1}$ matrix and their temporal patterns can be derived from the *S* matrix.

We used the time-domain extended Infomax ICA method to separate signal sources [19]. If an artifact is isolated in one of the independent components, then the inverse weights define a spatial fingerprint of the artifact, also called a topomap. Power spectra calculated from the *S* matrix define a component spectral fingerprint.

Inverse weights $W_{c}\left(n\right)$ were extracted from all n=1,2,…,N channels (representing topomaps). All weights $W_{c}\left(n\right)$ were arranged into a row vector. The subject matrix contains all c=1,2,…,C components. Subject matrices 35×256 (35 rows, 256 columns) were concatenated into one large 1890×256 data matrix *W* (rows = 35 component times 54 sessions; columns = 256).

The spatial extent of each component was emphasized by normalizing each row vector $W_{c}$ by its Root Mean Squared (RMS) value.

The dimension of the normalized matrix *W* was finally reduced by principal component analysis (PCA) to 1890×45. PCA reduction corresponds to standard preprocessing prior to dimension reduction with t-SNE.

We also calculated the power spectra $S_{xx}\left(f\right)$ from individual ICA time series contained in the *S* matrix. The power spectra were calculated from 0 to 100 Hz with steps of 1 Hz. The multi-taper method implemented in the FieldTrip toolbox was employed to stabilize the FFT estimation. Subject matrices 35×101 (35 rows, 101 columns) were concatenated into one 1890×101 data matrix (rows = 35 components × 54 sessions; columns = 101 frequency samples).

### 2.3. t-SNE Component Embedding

A nonlinear method was chosen to reduce the dimension. ISOMAP is one of the older approaches that has been superseded. For this reason, we chose the t-SNE method (described below).

The conditional probability $P_{i|j}$ represents the similarity between each pair of IC topomaps $W_{i}$ and $W_{j}$ (in the same way that IC power spectra $S_{i}$ and $S_{j}$ are similar) [20]. tSNE is based on the calculation of the conditional probability $P_{i|j}$ according to Equation (5).

(3) $$P_{i|j}=\frac{exp\left(-∥W_{i}-W_{j}{∥}^{2}\right)/\sigma_{i}^{2}}{\sum_{k\neq i}exp\left(-∥W_{i}-W_{k}{∥}^{2}\right)/\sigma_{i}^{2}},$$

where $\sigma_{i}$ is the variance of the *i*th component Gaussian distribution of neighbors. A particular value $\sigma_{i}$ corresponds to the perplexity hyperparameter, which has to be set by the user. The perplexity level influences the sensitivity of the algorithm to the local or global structure of the data. This parameter was experimentally set to different values (20, 30 and 40) to compare the effect on the result in 2D and 3D space. This is discussed in further detail in Section 3.

We applied the metric described in Equation (5) to two-dimensional space. Our two-dimensional space consisted of alternative distributions of topomaps/power spectra. The described algorithm iteratively minimizes the Kullback–Leibler divergence between high- and low-dimensional space probabilities (in our case, map/power spectra probabilities) [20]:

(4) $$KL\left(P||Q\right)=\sum_{i}\sum_{j}p_{i,j}log\frac{p_{i,j}}{q_{i,j}},$$

where $p_{i,j}$ is the symmetric conditional probability and $q_{i,j}$ is the probability of being obtained through Student’s t-distribution with one degree of freedom.

The minimum value of the Kullback–Leibler divergence KL is calculated using a gradient descent [20]:

(5) $$\frac{\delta \;KL}{\delta \;y_{i}}=4\sum_{j}\left(p_{i,j}-q_{i,j}\right)\left(y_{i}-y_{j}\right)\left(1+|\right|y_{i}-y_{j}{\left|\right|}^{2}{)}^{-1}$$

where $y_{i}$ and $y_{j}$ are low-dimensional counterparts of mass data points $W_{i}$ and $W_{j}$. Using one degree of freedom, the Student’s layout $\left(1+|\right|y_{i}-y_{j}{\left|\right|}^{2}{)}^{-1}$ decreases by the square of the distance for large paired distances $\left|\right|y_{i}-y_{j}\left|\right|$.

### 2.4. DBSCAN Clustering

Density-based algorithms create clusters using the spatial proximity of points and the change in this distribution [21]. The advantage of density algorithms is their ability to distinguish nested clusters (see Reference [21]). An alternative unsupervised algorithm is the K-means algorithm, although it requires the number of clusters as an input. In our study, we investigated the hidden internal structure of the data; as a result, we do not know the original number of clusters. In our case, the objects are the topographical maps and power spectra of IC signals. DBSCAN calculates the density of points, that is, the number of points in a predefined area (radius around the currently clustered point).

DBSCAN needs two input parameters—the radius (Eps) and the number of objects in it (k), which determine the density. Objects that constitute a specific density distribution are clustered into the same cluster and Eps determines the distance of points to be considered a part of a cluster. This means that if the Eps value is high, then we will reach a larger area; thus, the points may be more dispersed. *k* defines the number of points we need to create a dense area. The higher the value, the denser the area we look for and more bent points will be created. Eps and *k* are linked—if Eps is large, we can allow larger *k* values and vice versa.

The basic criterion for the size of *k* is the condition that *k* must be at least the size of the space dimension plus one. At the same time, if the data file is larger, the value of *k* should also be larger. On the basis of previous work with EEG space, we heuristically set the *k* value to 20. We used a k-dist sorted graph (k-plot) as a determiner of the Eps value. The k-plot is a weighted-directed graph, in which the *x*-axis represents points that are sorted by the nearest distance and the *y*-axis represents the distance for determining Eps. The ideal Eps corresponds to the “knee” in the k-plot. We determined that Eps = 7 in our reduced space.

The nested clusters are able to separate DBSCAN because the cluster boundary forms a site with a large density difference (high dot density region that is separated by a low-density location). All objects that are very distant (fall into a very low density area) are referred to as the noise cluster.

### 2.5. Applied Criteria

The relationships between the component data structure were quantified by calculating four criteria—the RMS energy of each component, autocorrelation, focal topography and focal trial activity [8]. These criteria were used to interpret each cluster of artifacts.

RMS energy describes energy dissipation (voltage). It is the square of the arithmetic mean of the squares of values (see Equation (6)).

(6) $$ERMS_{c}=\sqrt{\frac{1}{n}\cdot \left(X_{1}^{2}+X_{2}^{2}+\ldots +X_{n}^{2}\right)},$$

where *c* is a component and *X* is a time-series value.

In contrast to artifact components, the autocorrelation $A_{c}$ is assumed to reach high values in physiological ICs. The $A_{c}$ can be calculated as follows [8]:

(7) $$A_{c}=\sum_{t=l}^{T}x_{c}\left(t\right)\cdot x_{c}\left(t-l\right),$$

where *l* is the lag (set to 20 ms) for component *c* and $x_{c}\left(t\right)$ is time-domain data.

The criterion focal topography $F_{c}$ provides information about spatially focused energy in one or a few channels with the help of the maximum Z-score of energy across channels [8] (see Equation (8)).

(8) $$F_{c}=\max_{n}\left(\underset{N}{Z}\left(W_{c}\left(n\right)\right)\right),$$

where $\underset{N}{Z}$ denotes a set of Z-scores in all channels, $\max_{n}$ is the maximum in channel *n* and $W_{c}\left(n\right)$ is the inverse weight at channel *n* and component *c*.

Focal trial activity $FT_{c}$ measures the focality in time within the IC time series. Artifacts are indicated by a high signal amplitude that is not evenly distributed over time. The focal trial extraction is described in Equation (9).

(9) $$FT_{c}=\max_{k}\left(\underset{K}{Z}\left(\max_{t}\left(x\left(c,k,t\right)\right)-\min_{t}\left(x\left(c,k,t\right)\right)\right)\right),$$

where $\max_{t}$ is the maximum in a time sample *t*; $\min_{t}$ is a minimum in a time sample *t*; *k* denotes the *k*th segment of the signal; $\underset{K}{Z}$ is a set of Z-scores in all segments; and $x\left(c,k,t\right)$ are time-domain data in the *c*th component, the *k*th segment and time sample *t* [8].

In addition to the criteria described above, the RMS energy of each component was calculated to obtain more complete information. With the use of autocorrelation, focal trial, focal activity and RMS energy information, we were able to better evaluate the outcomes of our original methodology.

## 3. Results

We first present low-dimensional maps using the t-SNE method for IC spectral contents (spectral power) and IC spatial contents (topomaps). The structure of the spectral content is depicted to illustrate that there are frequency patterns in the data. The spatial content (topomaps) is also included in the scope of this paper for comparison with the previously published results. Different levels of perplexity were examined to ensure the robustness of the final mapping. The DBSCAN created clusters that were assumed to be the hidden patterns in the data. Finally, the found patterns were interpreted as neuroimages and further evaluated by presenting topographical maps, time-domain series, frequency-domain patterns and the commonly used EEG criteria described in Section 2. Our approach identified structures in hidden EEG layers; these structures corresponded to electrooculographic (EOG), electromyographic (EMG), electrocardiographic (ECG), gradient and line noise artifacts.

### 3.1. Space after t-SNE

**Figure 2:**
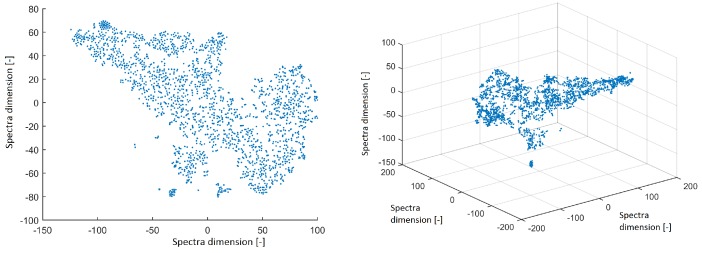
Reduced space of spectra to 2D from independent components (ICs) after using the t-distributed stochastic neighbor embedding (t-SNE) method with a perplexity of 30 (**left**). Reduced space of spectra to 3D from ICs after using the t-SNE method with a perplexity of 30 (**right**).

All power spectra were projected into 2D and 3D space using the t-SNE method with a perplexity of 30. The data structure is shown in Figure 2. The spectra from ICs created isolated clusters—this is a promising result for further investigation. However, we focus on the structure of IC topographic space in this paper to compare the findings in this study with the results obtained from EEG out of the MRI scanner in our previous study.

**Figure 3:**
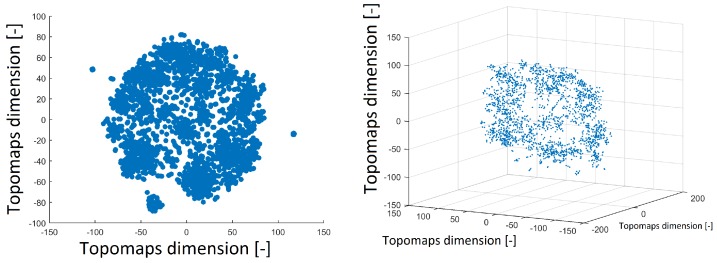
Reduced space of topomaps after using the t-SNE method to 2D space (**left**). Reduced space of topomaps after the using t-SNE method to 3D space (**right**). In both examples, a perplexity of 30 was used.

The t-SNE method was also used to project 1890 IC topomaps. These datasets were projected into 2D and 3D space by the t-SNE method, as depicted in Figure 3. The layout of 3D space was similar to that of 2D space. There was a cluster in the middle of the spherical space of the point and the other clusters formed the “mantle” of the sphere.

The perplexity parameter slightly affected the distribution of points after applying t-SNE. Thus, we varied the perplexity values (40, 30 and 20) to ensure the robustness of the results. A perplexity of 30 was used in the previous study [15].

**Figure 4:**
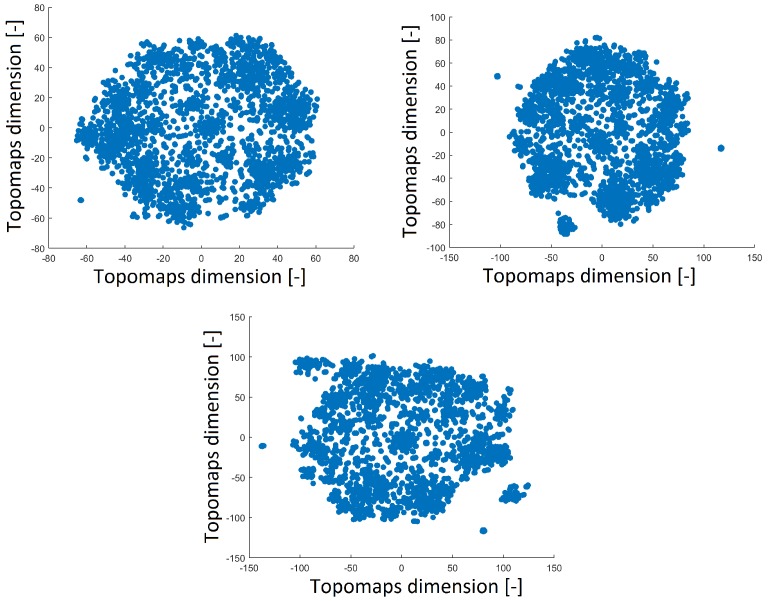
Reduced space of topomaps after using t-SNE with a perplexity of 40 (**top left**). Reduced space of topomaps after using t-SNE with a perplexity of 30 (**top right**). Reduced space of topomaps after using t-SNE with a perplexity of 20 (**bottom**). For all settings, the perplexity was used to reduce IC space.

A perplexity of 20 reduced the space with a distribution that was opposite of that obtained with perplexities of 30 and 40, as shown in Figure 4. The distribution of the 2D projection with a perplexity of 30 was similar to that of the projection with a perplexity of 40 (a high perplexity has a compact distribution with a minimum number of outlier clusters). The number of resulting clusters can be observed in Table 1. The smallest perplexity led to fewer clusters and more noise points, as shown in Table 1. However, we found that the most significant patterns were preserved across all perplexity values, as we describe in Section 3.3. Thus, we fixed the perplexity level to 30 for comparison with our previous results.

### 3.2. DBSCAN Clustering

**Figure 5:**
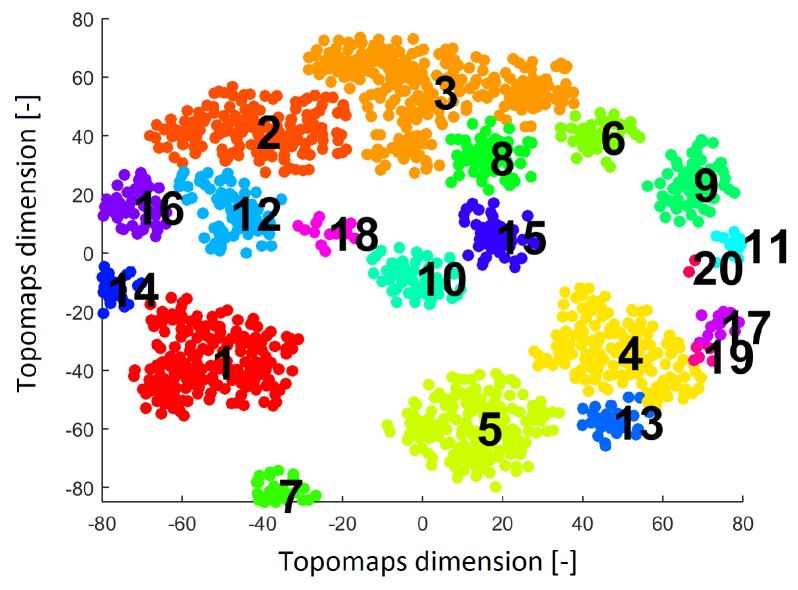
Result after applying the clustering algorithm Density-based spatial clustering of applications with noise (DBSCAN) to the IC space resulting from the t-SNE method with a perplexity of 30. The result shows 20 clusters (each cluster is labeled by a number and is a different color).

The DBSCAN algorithm was applied to the t-SNE maps for all values of perplexity. We focused on the IC space of topomaps (not spectra) since many others studies have shown interesting results in the analysis of the spatial content of components (for example, References [8,15,22]). The distributions resulting from perplexities of 30 and 40 led to a similar number of noise points and the same numbers of clusters (see Figure 5); this result confirms the qualitative comparison in Section 3.1.

Twenty clusters labeled without noise points formed by DBSCAN are shown in Figure 5. Each cluster contains ICs originating from various subjects.

### 3.3. Meaning of the Clusters

The median and mean (for every DBSCAN cluster) of the topomaps, Fourier spectra and time series are examples of the importance of the clusters found. In clustering, clusters generally do not need to be meaningful. Some artifacts are visually observable in the spectrum, while others are in the time domain or topomaps. For this reason, only the images that are typical of each artifact are used below.

**Figure 6:**
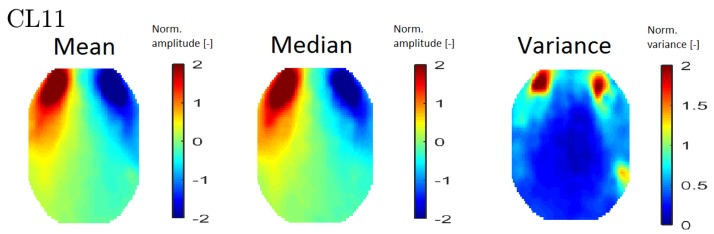
An example of all map types—mean topomaps from cluster 11 (**left**), median topomaps from cluster 11 (**middle**) and variance topomaps from cluster 11 (**right**).

In order to interpret the results, an averaged topomap was calculated for each cluster. Topomaps that represent the median and variance values within each cluster were also calculated. The median and mean topomaps are qualitatively similar, which indicates cluster homogeneity (see Figure 6 (left) and (middle)). An example of a variance map can be seen in Figure 6 (right).

**Figure 7:**
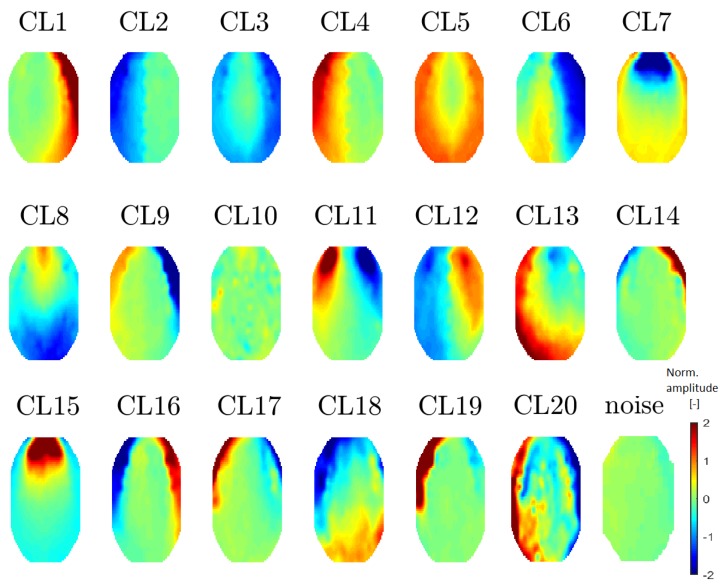
Mean topomaps representing 20 clusters after using the t-SNE method with a perplexity of 30. A perplexity of 40 had the same topomaps that differed in sequence. The mean and median topomaps have similar characters but the variance has no meaningful value. Topomaps were clustered from IC space.

**Figure 8:**
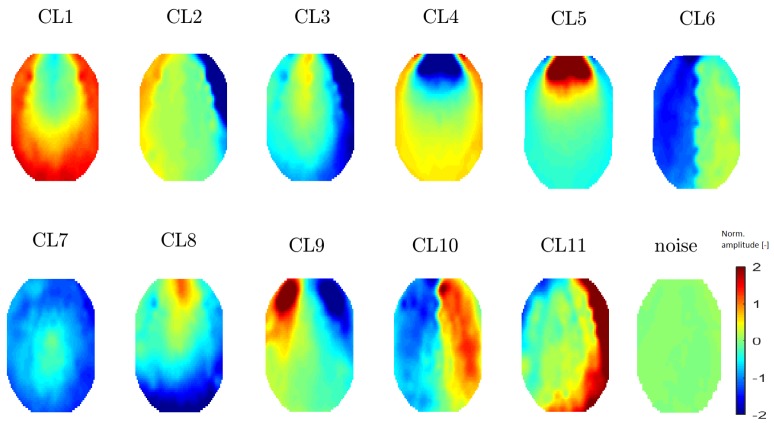
Mean topomaps representing 11 clusters after using the t-SNE method with a perplexity of 20. The mean and median topomaps have similar characters but the variance has no meaningful value, similar to the case with perplexities of 30 and 40. Topomaps were clustered from IC space.

All 20 topomaps representing 20 determined clusters (using a perplexity of 30 or 40) are presented in Figure 7. All 11 topomaps representing 11 clusters determined after using t-SNE with a perplexity of 20 are depicted in Figure 8. Clustering with t-SNE with the perplexity set to 20 did not present any extra benefit compared with a perplexity of 30. For this reason, we focused on a t-SNE perplexity of 30.

A perplexity of 30 was also used in our previous work. In that study, we processed data that were measured in Faraday’s cage with a bandpass filter of 0.5–50 Hz. Our previous method resulted in 12 clusters (see Reference [15]). A higher number of clusters was found in this work because of the higher number of artifacts (for example, gradient artifact; see Section 3.3.3). In the following sections, we explore the meaning of the clusters (identify clusters that represent certain EEG artifacts) on the basis of topomap, spectral and time-domain analysis.

#### 3.3.1. EOG Artifact

**Figure 9:**
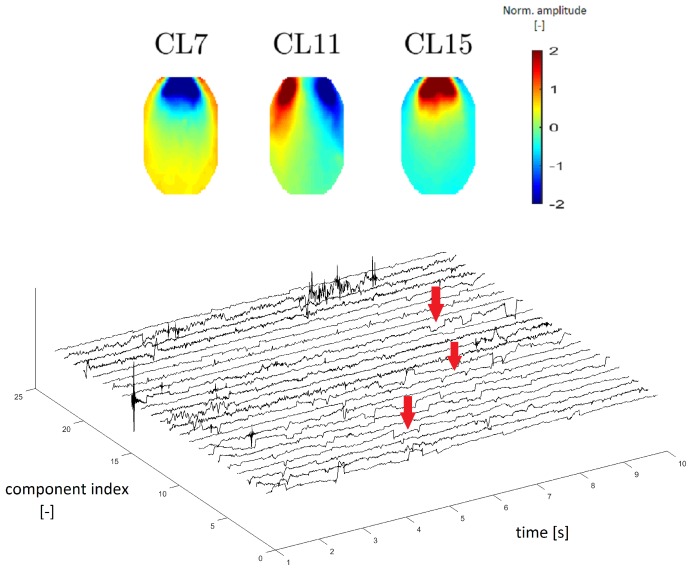
Topomaps of the clusters representing electrooculographic (EOG) artifacts from different eye activity (**top**). Time-series graphs of a 10-s section of the signal that were clustered into the same cluster by IC topomaps (**below**). Arrows point to an example of the EOG artifacts, which are manifested as “rectangular steps” in the time-series graphs.

In accordance with Reference [8], we estimated that clusters CL7, CL11 and CL15 represent EOG artifacts, which represent eye movements and blinking. Figure 9 (top) depicts an example of different eye movements (EOG artifacts). Different eye movements have specific topomaps. An upward movement of the eyes shows a very pronounced gain in the frontal area, which gradually decreases occipitally [23]. This transition is observable in CL15 in Figure 9, in which the topomaps have the same described character as those in Reference [23]. The topomap has an inverted character (opposite of CL15) when the eye moves downward (see the topomap of CL7 in Figure 9). The topomap of CL11 corresponds to eye movement from right to left, with a significant increase in the left frontal area and a significant decrease in the right frontal area [23].

EOG artifacts are manifested by slow waves of higher amplitude in time series. In Reference [24], we verified this claim using time-series graphs. The “steps”, as shown in Figure 9 below, are typical of an eye artifact in a time series.

EOG topographic maps, frequency spectra and time-domain signals were described in Reference [25]. In Reference [25], we verified that the mean power spectrum of EOG artifacts corresponded to the one observed. The EOG artifact typically contains the main power at low frequencies, which was also observed in our mean power spectrum. Eye movements (horizontal, vertical and blinking) are also comparable to results in the literature ([24,25]).

#### 3.3.2. EMG Artifact

**Figure 10:**
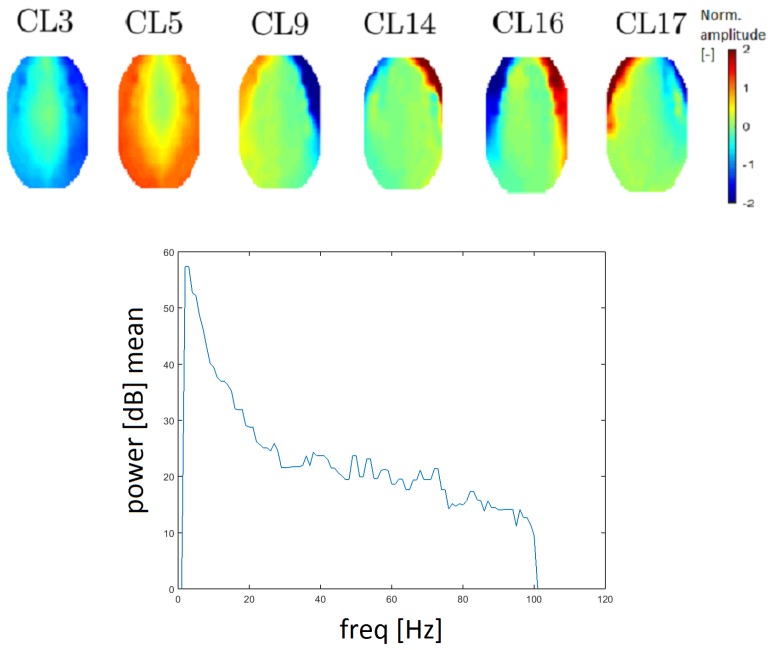
Topomaps of clusters that represent electromyographic (EMG) artifacts (**top**). The mean power spectrum after median filtration of the CL9 cluster, which represents EMG artifacts (**bottom**).

EMG artifacts (muscle artifacts) are manifested across the spectrum and topographic maps corresponding to individual narrower spectral values of muscle activity have a variable character [26,27]. It follows from Reference [28] that the shift of activity moves from the frontal to the occipital area for higher frequencies. EMG artifacts are reflected by higher power, especially in the edge electrodes [26]. Clusters CL3, CL5, CL9, CL14, CL16 and CL17 (see Figure 10) correspond to the definition of an EMG artifact. There is an apparent transition of activity from the frontal to the occipital area. The topomap in clusters CL3 and CL5 contain dominant muscle activity that could be caused by mastoidea or ear movements.

The power spectrum has a higher value for higher frequency (compared with other clusters). EMG artifacts are mostly defined by the low edge of a frequency spectrum (from 20 Hz) and an example of a frequency spectrum is provided in Figure 10. Median filtration was used to smooth the power spectrum in order to inspect higher frequencies. Therefore, the results are more easily interpreted. The spectrum for a muscle artifact is higher (above 20 dB) for higher frequencies opposite, for example, to the line noise artifact spectrum, which is around 10 dB.

#### 3.3.3. Gradient Artifact

A gradient artifact (GA) is an artifact from the gradient coil of the fMRI. A GA has a periodic character in the spectrum; it resembles a “rake”, with an initial peak at low frequency (15–20 Hz), followed by its higher harmonics (see Reference [16]). GA residues were found in the spectra that correspond to clusters CL2, CL4 and CL10. We evaluated the spacing of individual local maxima in the power spectrum and we found that the spacing was constant at 18 Hz between higher harmonic frequencies. In this case, the spectrum was computed without median filtration because the spectral lines representing the GA are narrow.

**Figure 11:**
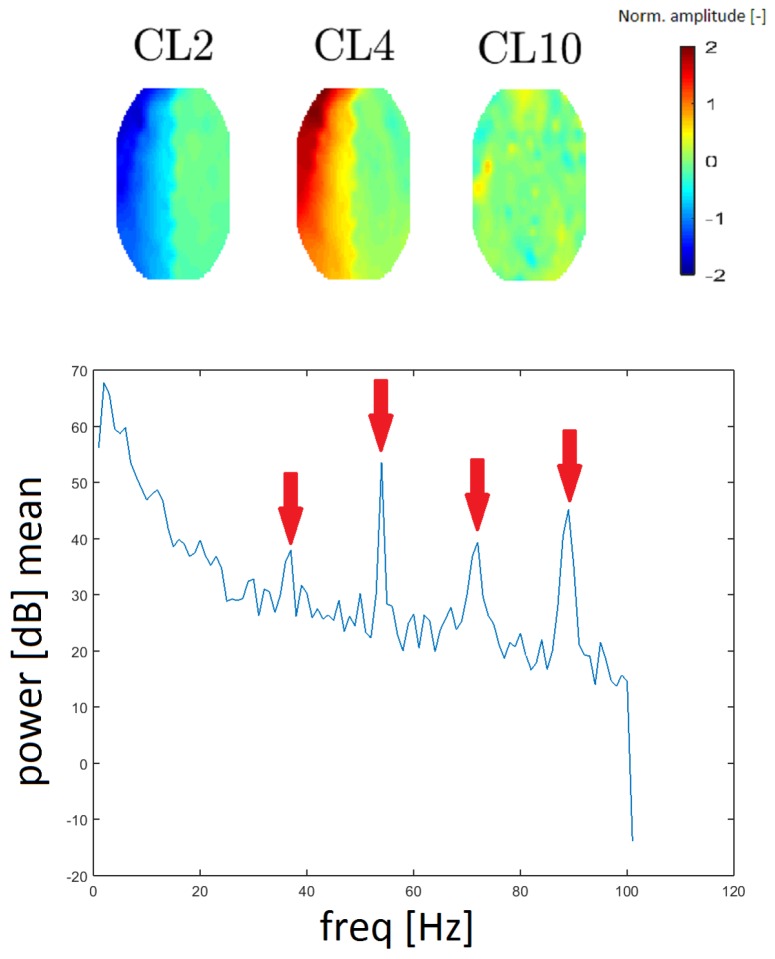
Topomaps of the clusters representing gradient artifact (GA) residues from magnet coil switching (**top**). The mean power spectrum of the GA found for cluster CL10 (**bottom**). Arrows show the amplitudes at 36, 54, 72, and 90 Hz (spacing of 18 Hz). The mean power spectra for clusters CL2 and CL4 are similar in appearance.

To the best of our knowledge, topomaps of GAs have not been published. The topomaps of the clusters CL2 and CL4 are the inverse of one another (they likely originate from the same principal component). Although the topomap of cluster CL10 differs in shape (see Figure 11), its frequency response is the same as that of CL2 and CL4 described above. Thus, we assume that it also represents the GA artifact. The reason that CL10 has a different spatial representation is a question that needs further exploration.

In the time series, we did not visually find a standard manifestation of a high-amplitude GA artifact. Thus, the residue is not evident at first sight during clinical scoring.

#### 3.3.4. ECG Artifact

A topomap of an ECG artifact (artifact that represents heartbeats) is shown in Reference [24]. In this study, the ECG topomap is represented by descending performance across the head. Cluster CL18 has a similar topomap character.

**Figure 12:**
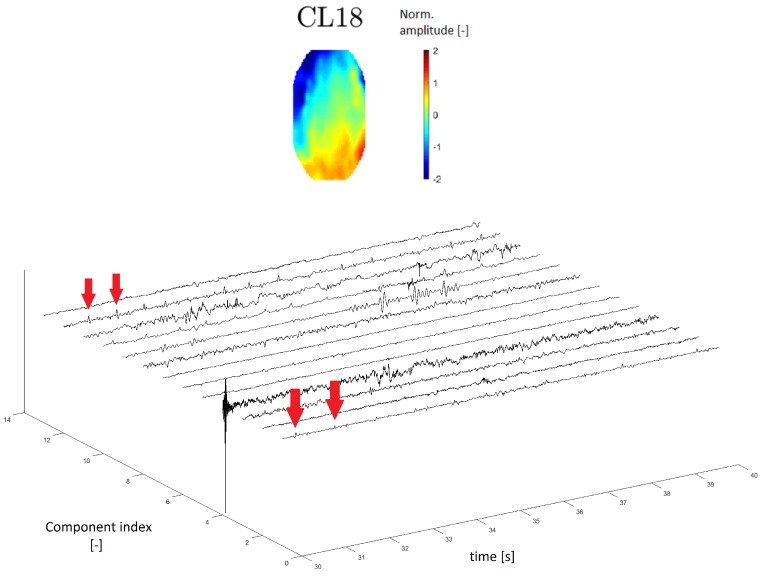
Topomap of cluster CL18 representing an electrocardiographic (ECG) artifact (**top**). Time-series graphs of a 10-second section of the signal clustered into the same cluster by IC topomaps. The arrows indicate an example of the ECG peak residuum, which manifested in the time-series graphs.

In the time series, the ECG artifact manifested as a sharp peak that occurred at regular intervals of the frequency of heartbeats. The time series of cluster CL18 contains residual peaks with a frequency of about 1 Hz (see Figure 12), which is the approximate frequency of heartbeats.

In the power spectrum, the frequency of the ECG artifact is hidden in physiological activity, which has significant power at lower frequencies of 0.5–4 Hz (delta band).

#### 3.3.5. Line Noise Artifact

**Figure 13:**
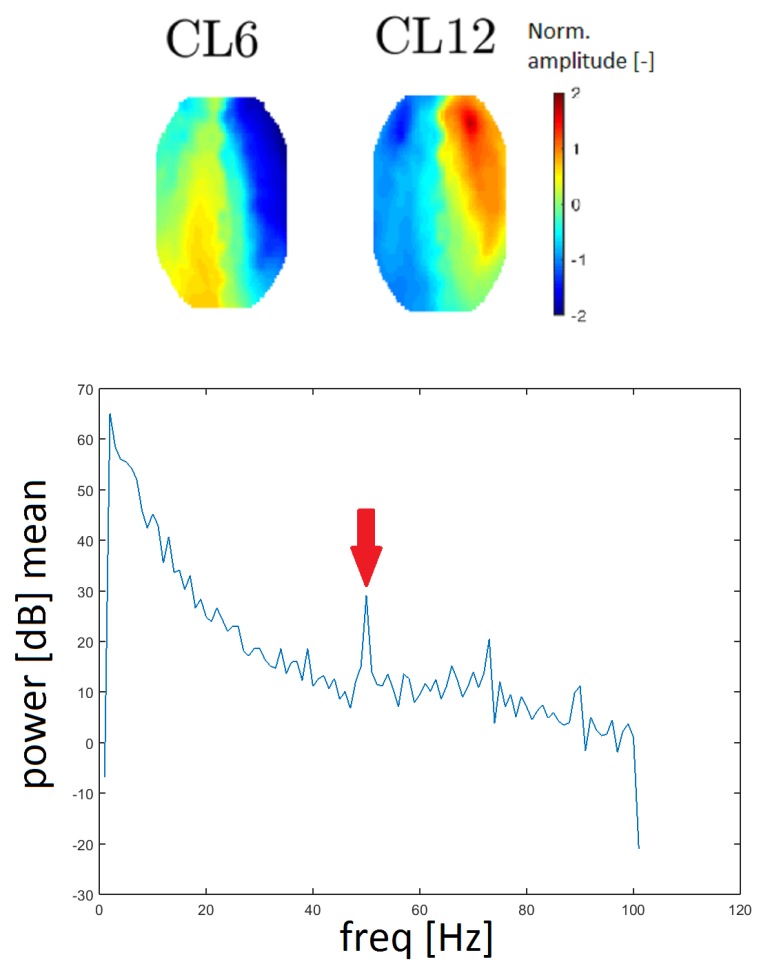
Topomaps of clusters CL6 and CL12, which have a distinctive peak at a frequency of 50 Hz, as line noise artifacts (**top**). The mean power spectrum of cluster CL6 with a distinctive peak at a frequency of 50 Hz (arrow).

Line noise is an artifact caused by the socket of electrical outlets (with a frequency of 50 Hz in Europe). Two clusters, CL6 and CL12, had a very distinctive peak at that frequency in the spectrum and they also had similar topomaps (see Figure 13). In this case, the spectrum was computed without median filtration because the band of a line noise artifact is very narrow.

#### 3.3.6. Undefined Clusters

It is worth noting that the proposed technique searches for clusters without any prior assumption about their meaning. Thus, not all regularities found in the data represent information that is relevant to the question asked.

It is expected that undefined clusters consist of a combination of noise, outlying points, artifacts of the embedding method, unknown types of artifacts and most importantly, physiological activity.

The noise group contains outlying points, which DBSCAN formed into a single noise group. This group is not considered to be a cluster. Instead, it is a region of points that is likely due to intra-individual variability, individual recording settings and parameters that vary among studios. Thus, all recordings differ in this aspect. It is a set of components without a structure. This structure cannot be mixed with components.

Five undefined clusters do not reflect the nature of any known artifact. These types of clusters could be related to physiological activity. We emphasize that the clustering method itself is the most probable origin of undefined clusters. As mentioned above, the nonlinear dimension reduction procedure is capable of fitting data patterns that are unrelated to physiological or artifact activity. It is also possible that a separate cluster was formed by an artifact that has not yet been clinically described in the EEG field.

### 3.4. EEG Criteria

**Figure 14:**
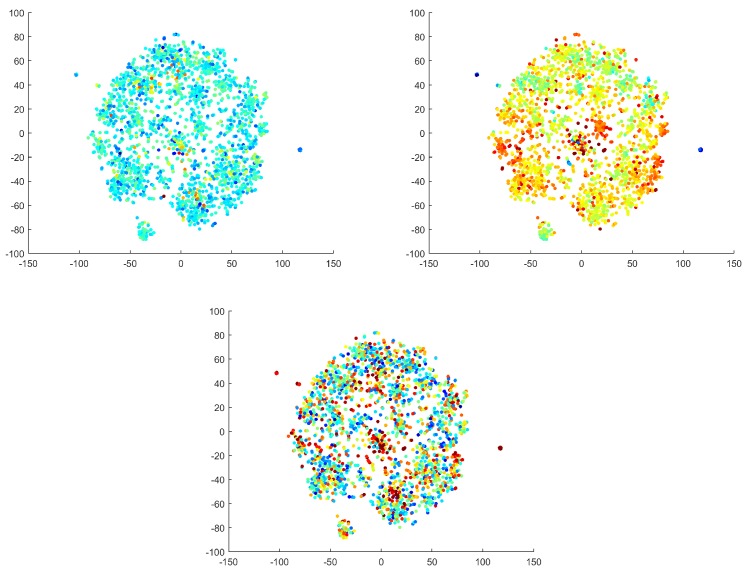
Commonly used EEG criteria displayed in IC space. The autocorrelation criterion on a logarithmic scale (**top left**); the focal criterion on a logarithmic scale (**top right**); the focal trial criterion on a non-logarithmic scale (**below**). The dimensions are formed by topomaps and are dimensionless.

The selected criteria are commonly used to describe IC space and distinguish artificial sources from others [8]. We calculated the criteria for our ICs and used the values to label the reduced 2D IC space using a color gradient (see Figure 14). It was necessary to convert two of the criteria to a logarithmic scale to highlight the differences (color transitions). The focal trial and logarithmic focal have characters that are nearly the inverse of one another. Autocorrelation has a few distinct points (with different values); otherwise, this criterion does not significantly partition the space. The predefined criteria were utilized to describe the found clusters by quantitative metrics from the literature. The criteria were used to interpret the found clusters by features other than their spectral and spatial patterns. We emphasize that the manifold learning and clustering procedures were not informed by the criteria. Moreover, we found that the qualitative interpretations of clusters were not always consistent with the predefined criteria.

## 4. Discussion

The present study focus on the exploration of the spatial properties of independent components. Nevertheless, we also briefly present their spectral properties. In this regard, we only evaluated whether our dataset contained spectral patterns that could be used to inform IC classifiers. From the obtained spectral maps, we conclude that it will be valuable to focus on joint mapping (spatial and spectral) in our future research on nonlinear dimension reduction techniques.

Our main research goal was to explore the topomap space as it evaluated a large number of EEG studies. We confirm that the layout in 2D space after applying t-SNE has a similar character to that in 3D space after t-SNE and the clusters form circles in 2D space and spheres in the 3D space. We found that the 2D representation was appropriate for visualizing topomaps and spectral spaces.

We also studied the effect of the perplexity parameter on the resulting layout in 2D space. For smaller values of perplexity (20), several small outlying clusters were produced in the space and this led to more noise points during DBSCAN clustering. A perplexity of 30 appears to be an ideal setting since higher values of perplexity (40) do not significantly change the layout in 2D space. Moreover, the shapes of the resulting clusters are the same as those of the topomaps. Finally, we found that changing the value of perplexity (when varied in a reasonable range) does not affect the final output.

For the goal of this study, DBSCAN can be used as a linear clustering technique (linear metric for distance calculation) since the t-SNE method translates even complex manifolds to compact, low-dimensional distributions. It is important to mention that DBSCAN was also applied to the original high-dimensional dataset and no interpretable results were obtained.

We set Eps to 6 and the number of objects to 20 because these values correspond to the ideal distribution of the midpoint/noise ratio in the resulting clustering and correspond to the number and distribution of the clustered points in 2D space. We used the clustering to divide the data in the original multidimensional space so that we could use the least distorted data to compute the topomaps. We sought patterns corresponding to individual artifacts (EOG, EMG, ECG, gradient and line noise) in the map and the spectrum.

EOG has a very clear topomap structure for vertical movements, horizontal movements and blinking, and the maps representing eye artifacts appear to be clearly defined. Their occurrence is also confirmed by a lower spectral power for higher-frequency values.

There are other options for suppressing the EOG artifact. Our methodology could be a suitable criterion for comparing the effectiveness of ophthalmic artifact suppression using various algorithms [29]. Generally, since eye activity has a high amplitude in the original EEG time series, the EOG artifact is involved in all three modalities (topomap space, frequency and time). This assumption is supported by our previous studies [22,24,25,30].

EMG artifacts derived from cranial muscles are diverse and have different spectral characteristics and this diversity makes identifying an EMG artifact difficult. We identified six clusters that likely represent EMG artifacts. We compared the character of the clusters in accordance with References [26,27] and found that they have two types of topomaps. The first type reflects distinctive activity that starts in the frontal region and continues to the temporal region. The second type of topomap for this cluster has distinctive activity in the majority of the space, except for the central region. These topomaps agree with results in the literature [28]. All EMG clusters have a higher power spectrum at higher frequencies and the power for cluster CL17 at higher frequencies declined faster than that of other EMG clusters. Combined with its topomap, cluster CL17 is identified as representative of EMG artifacts.

The gradient artifact can be suppressed by a standard method. However, our method was able to cluster the residuals from the GA artifact. We were unable to find a topographic map in the literature that corresponded to the gradient artifact, so we compared the average spectrum of our clusters with those of other studies [16]. We found peaks at higher harmonic frequencies (36, 54, 72 and 90 Hz) in all three averaged spectra. This spacing corresponds to 36 slices of magnetic resonance at a 2-s T setting (all determined by settings slices and repetition time (TRs) of fMRI). Therefore, we looked at the individual spectral characteristics of all the topomaps and we discovered three maps whose spectra had a clear GA pattern. Two of these maps (CL2 and CL4) have inverse shapes and they are very regular. In the middle of topomaps, the amplitude changes rapidly. This reflects the nature of a GA since it has a very high amplitude and a signal that reaches both positive and negative values. The CL10 cluster is rather similar to the topomap, which has no significant activity. According to the spectra, we confirm that our methodology identified the GA residue and divided it into three clusters.

Generally, a topomap of ECG activity has a typical crossing shape and the topomap of cluster CL18 is similar to that in the literature [24]. The cluster time-domain series exhibits residuals with a frequency of around 1 Hz, which is the approximate frequency of a heart beating.

The power spectra of clusters CL6 and CL12 present distinctive peaks at a frequency of 50 Hz, which represents a line noise artifact. The 50 Hz signal in the power spectra is also observed for other clusters but CL6 and CL12 exhibit much higher power at a frequency of 50 Hz. Furthermore, the topomaps of clusters CL6 and CL12 are similar to each other. These findings indicate that the line noise artifact has a significant influence on these clusters.

We extracted five clusters that do not correspond to known artifacts. It is possible that these artifacts have not yet been described or they may be generated by equipment operation in the recording room. They could also be physiological background or artifacts of the clustering methods since one of the properties of clustering methods is that they can produce artifacts themselves. In this case, these clusters may contain information that is irrelevant to our research.

The criteria used by the EEG standard were calculated from the components before the reduction of the dimension and we explored whether any criterion would separate one artifact from the others. From the space labelled by autocorrelation, we are unable to distinguish any structure. In general, we can only say that components have lower autocorrelation values up to exceptions.

The outlying clusters for the focal and focal trial criteria have inverse characters. The focal criterion is larger for the eye movement and gradient artifact clusters in the middle of the space. A larger focal criterion was found for the GA with high activity in topomap CL4 and a lower focal criterion was found for lower activity in topomap CL2. The opposite focal criterion values, therefore, correspond to the contradictory nature of the GA topomaps of CL2 and CL4. Overall, the standard EEG criteria did not help us to divide the original space.

## 5. Conclusions

In this work, we analyzed hdEEG datasets acquired during fMRI scanning. Generally, the combination of these two modalities generates a number of problems, including a new type of artifact in EEG signals. We present an unsupervised method that captures the imprint of the dataset at hand. First, hdEEG data are preprocessed (supervised section) and then ICs are computed. Then, the nonlinear t-SNE method reduces the resulting multidimensional space to 2D space, which is partitioned by the density-based clustering algorithm DBSCAN. Our results show that the proposed approach was capable of distinguishing various artificial components in the recordings—EOG, EMG, ECG, gradient and line noise artifacts were all distinguished with our methodology. The performance of our method was compared with an approach based on common criteria (features) and the criteria were not capable of isolating artificial components. Thus, we conclude that our exploration approach outperforms the feature-based method.

In future work, joint spatial and spectral mapping will be addressed. Our goal is to develop a tool that is capable of rapidly checking the quality of the dataset at hand, automatically perform IC clustering by learning the t-SNE map and learning from an online database with semiautomatic evaluation. The first step is to explore the typical structure of hdEEG and hdEEG/fMRI datasets.

In general, the inverse weights of the ICA algorithms (topomaps) do not provide information about patients or healthy individuals and thus do not introduce ethical problems. Thus, such topomaps, similar to images, can be shared between neuroscientists and a large database of patterns can ultimately be obtained and learned by a classifier.

## Figures and Tables

**Table 1:** The number of noise points and number of clusters resulting from DBSCAN for different values of perplexity in the *t*-SNE method used on IC space.

Perplexity [-]	No. of Clusters [-]	No. of Noise Points [-]
20	11	1678
30	20	525
40	20	858

## Abbreviations

The following abbreviations are used in this manuscript:

EEG	Electroencephalography
fMRI	Functional magnetic resonance imaging
DBSCAN	Density-based spatial clustering of applications with noise
ICA	Independent component analysis
PCA	Principal component analysis
tSNE	t-distributed stochastic neighbor embedding
GA	Gradient artifact
PA	Pulsion (cardioballistic) artifact
